# Behind the Counter Danger: Pseudoephedrine-Induced Seizure in an Elderly Patient

**DOI:** 10.7759/cureus.67099

**Published:** 2024-08-17

**Authors:** Fawzi Mudarres

**Affiliations:** 1 Internal Medicine, Hamad Medical Corporation, Doha, QAT

**Keywords:** treatment, flu, elderly, seizure, pseudoephedrine

## Abstract

In this case report, the potential of pseudoephedrine to induce seizures as a side effect in elderly patients is discussed. Pseudoephedrine is commonly used to relieve nasal congestion caused by allergies, colds, or sinusitis. Still, it has been linked to several side effects, such as cardiovascular events, nervous system toxicity, and drug interactions. The report presents the case of an 83-year-old man with a medical history of diabetes, hypertension, and dyslipidemia who experienced a generalized tonic-clonic seizure after taking an over-the-counter medication containing loratadine and pseudoephedrine. The discussion underscores the need for caution when prescribing pseudoephedrine to elderly patients, particularly those with a history of seizures or other neurological conditions, and recommends alternative treatments like nasal saline irrigation or other decongestants. The case highlights the importance of comprehensive neurological assessment and diagnostic testing in patients with seizures to identify the underlying cause and guide appropriate management. Additional research is necessary to better understand the potential risks and benefits of using pseudoephedrine in elderly patients and explore alternative options for treating nasal congestion.

## Introduction

Pseudoephedrine, a sympathomimetic agent, is widely utilized as a decongestant for the relief of nasal congestion resulting from allergies, colds, or sinusitis. Despite its efficacy, the use of pseudoephedrine has been linked to a spectrum of adverse effects, including cardiovascular events, nervous system toxicity, and drug interactions [1]. These adverse effects are particularly worrisome in elderly patients, who are more susceptible to adverse drug reactions (ADRs) due to age-related alterations in pharmacokinetics and pharmacodynamics, the presence of multiple comorbidities, and polypharmacy [2]. In the geriatric population, pseudoephedrine use can elevate the risk of cardiovascular complications such as hypertension (HTN), arrhythmias, and myocardial infarction, as well as nervous system toxicity manifesting as agitation, confusion, and seizures [3].

This case report seeks to underscore the potential of pseudoephedrine to induce seizures as an adverse effect in elderly patients. Although seizures are a rare but documented side effect of pseudoephedrine, it is crucial to acknowledge their occurrence, particularly in patients with pre-existing neurological conditions or those on medications that reduce the seizure threshold. Our report aims to heighten awareness among healthcare professionals regarding this potential risk and emphasize the necessity for meticulous monitoring when prescribing or recommending pseudoephedrine to elderly patients.

## Case presentation

An 83-year-old male with a history of type II diabetes mellitus managed with oral hypoglycemic agents (metformin), HTN treated with amlodipine, and dyslipidemia managed with atorvastatin presented to the emergency department (ED). The patient reported no past medical history of seizure disorder. He presented with upper respiratory tract symptoms, including a runny nose, sneezing, and a sore throat. He had self-prescribed an over-the-counter medication containing loratadine 5 mg and pseudoephedrine 120 mg for symptom relief at home. Following the ingestion of the first tablet at bedtime, he went to sleep. His wife reported finding him snoring loudly, then falling to the floor, feeling dizzy, and involuntarily urinating. No convulsions were witnessed at this time. The estimated time of the incident after pill ingestion was about four to six hours. He was brought to the ED by his son and was oriented upon arrival, with stable vital signs. 

However, during observation in the ED, he suddenly developed his second seizure, which was described as a generalized tonic-clonic seizure with tongue bite and loss of sphincter control. The episode lasted for one to two minutes and aborted spontaneously. Following this, he was given intravenous (IV) levetiracetam as a loading dose of 1 gm, then initiated on a maintenance dose of 500 mg twice daily (BID) during hospitalization.

On physical examination, he was drowsy in a postictal state but had no focal neurological deficits. During questioning, he reported no history of any substance abuse and was not on any herbal supplements. He was subsequently admitted for further evaluation. Laboratory tests (Table 1) were unremarkable, with a normal electrolyte panel. Point-of-care (POC) glucose was 90 milligrams per deciliter (mg/dL). An electrocardiogram (EKG) done in the ED revealed sinus rhythm with no other anomalies.

**Table 1:** Laboratory tests

Labs	Values (reference values)
White Blood Cell Count (WBC)	6,000 /µL (4-10 x 10^9/L)
Hemoglobin (Hgb)	10.1 g/dL (12-16 g/dL)
Platelets (Plts)	168,000 /µL (150,000-350,000/µL)
Urea	24 mg/dL (8-20 mg/dL)
Creatinine (Cr)	1.04 mg/dL (0.8-1.3 mg/dL)
Sodium (Na)	136 mEq/L (136-145 mEq/L)
Potassium (K)	3.5 mEq/L (3.5-5.0 mEq/L)
Calcium (Ca)	2.17 mmol/L (2-2.6 mmol/L)
Magnesium (Mg)	0.73 mmol/L (0.85-1.10 mmol/L)
Glucose	96 mg/dL (70-99 mg/dL)

**Figure 1:**
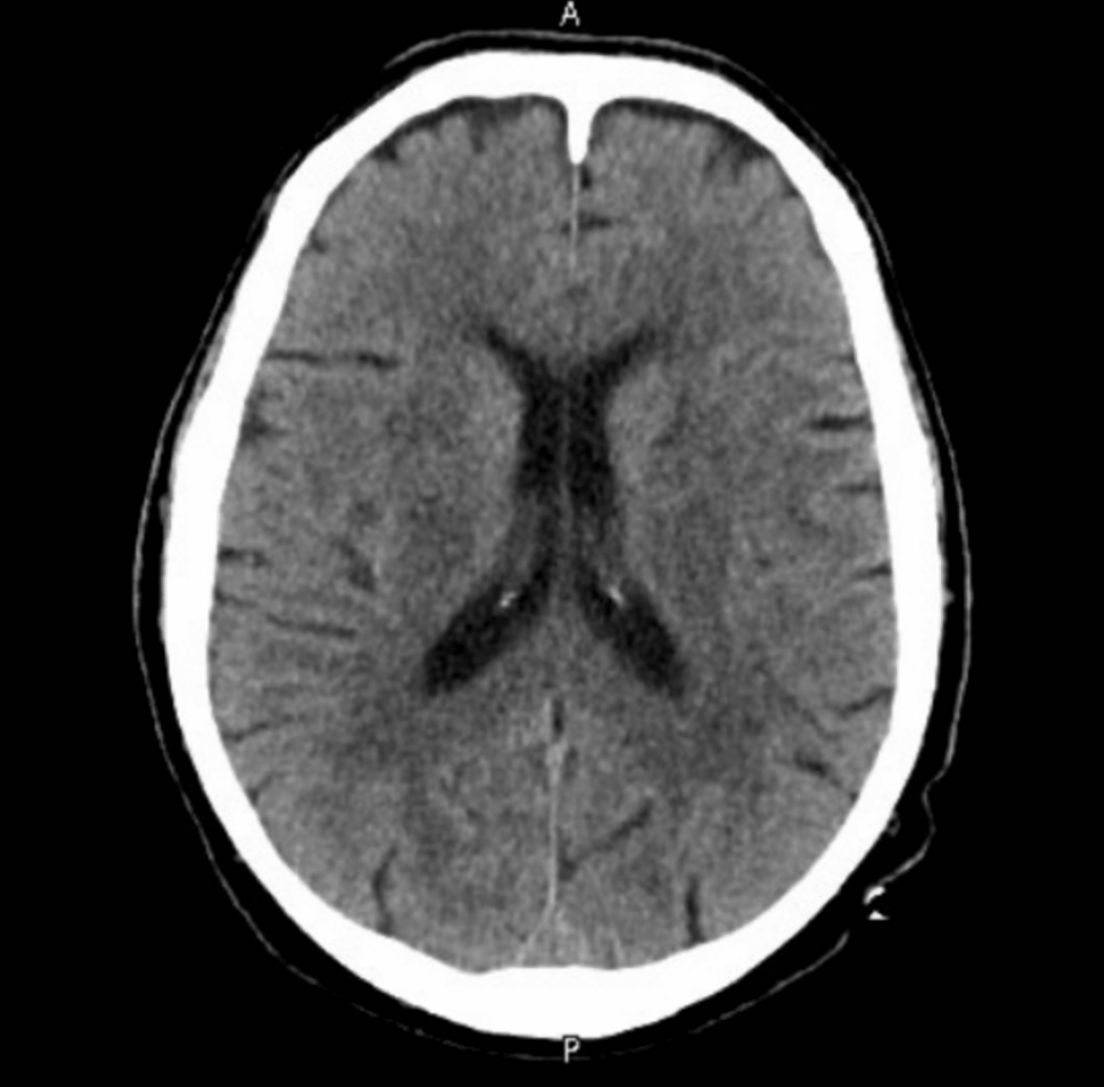
CT head section 1

**Figure 2:**
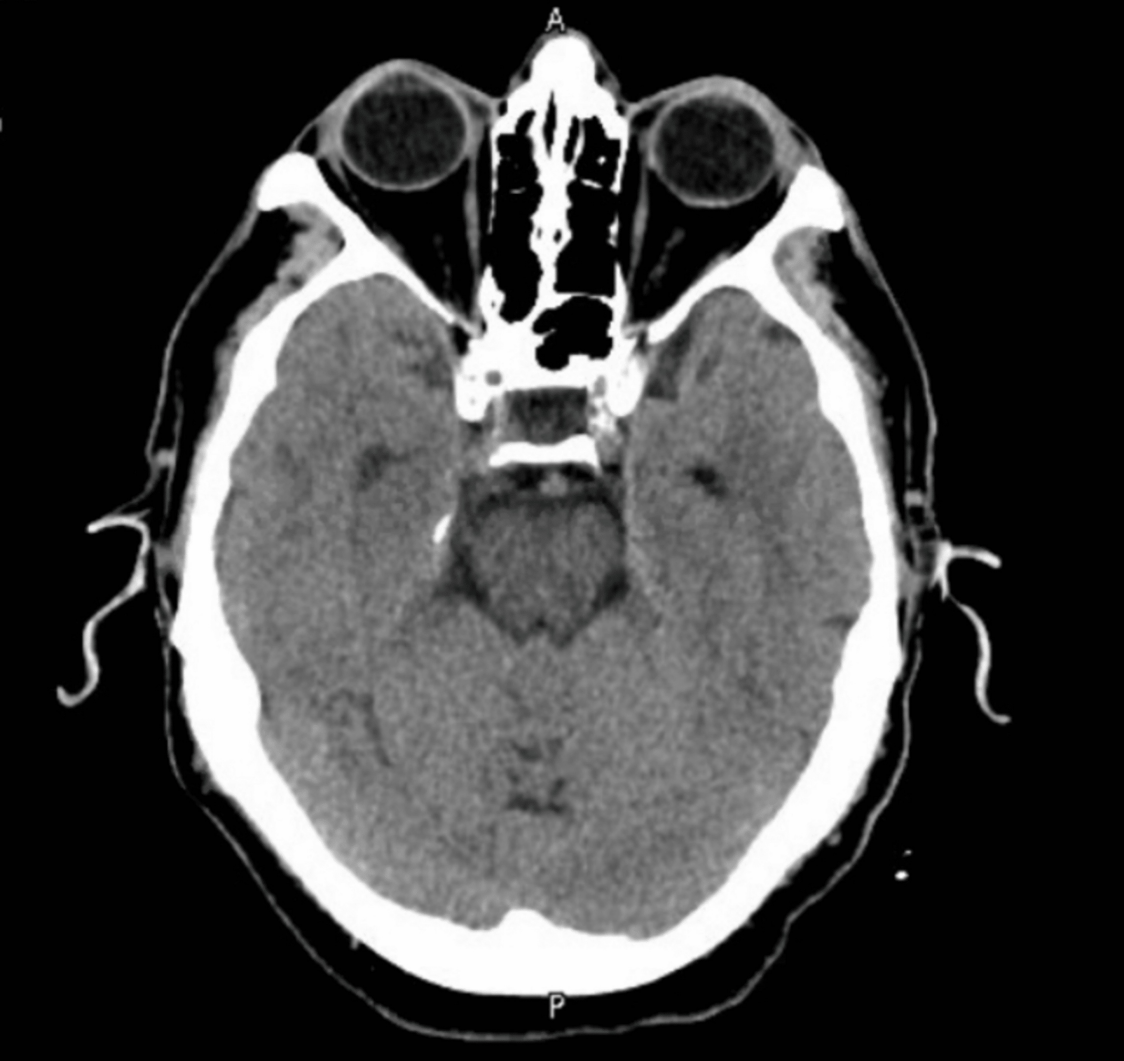
CT head section 2

**Figure 3:**
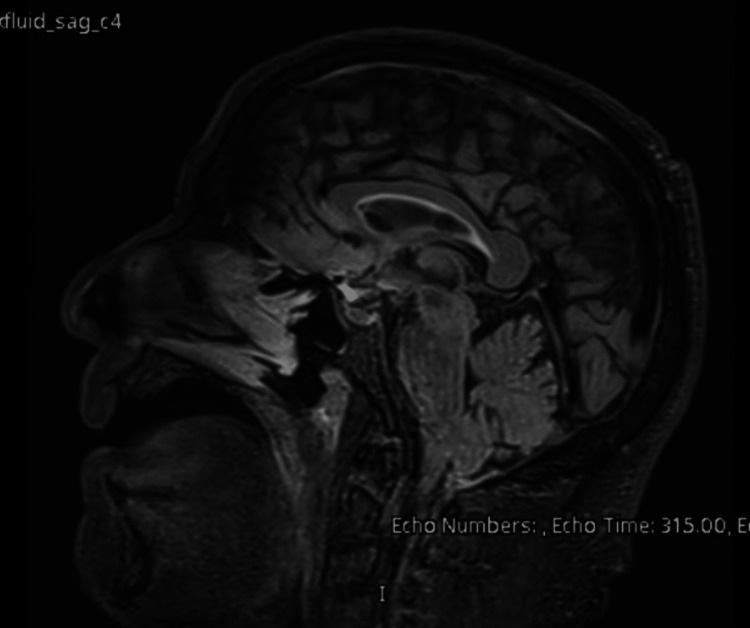
MRI head section 1

**Figure 4:**
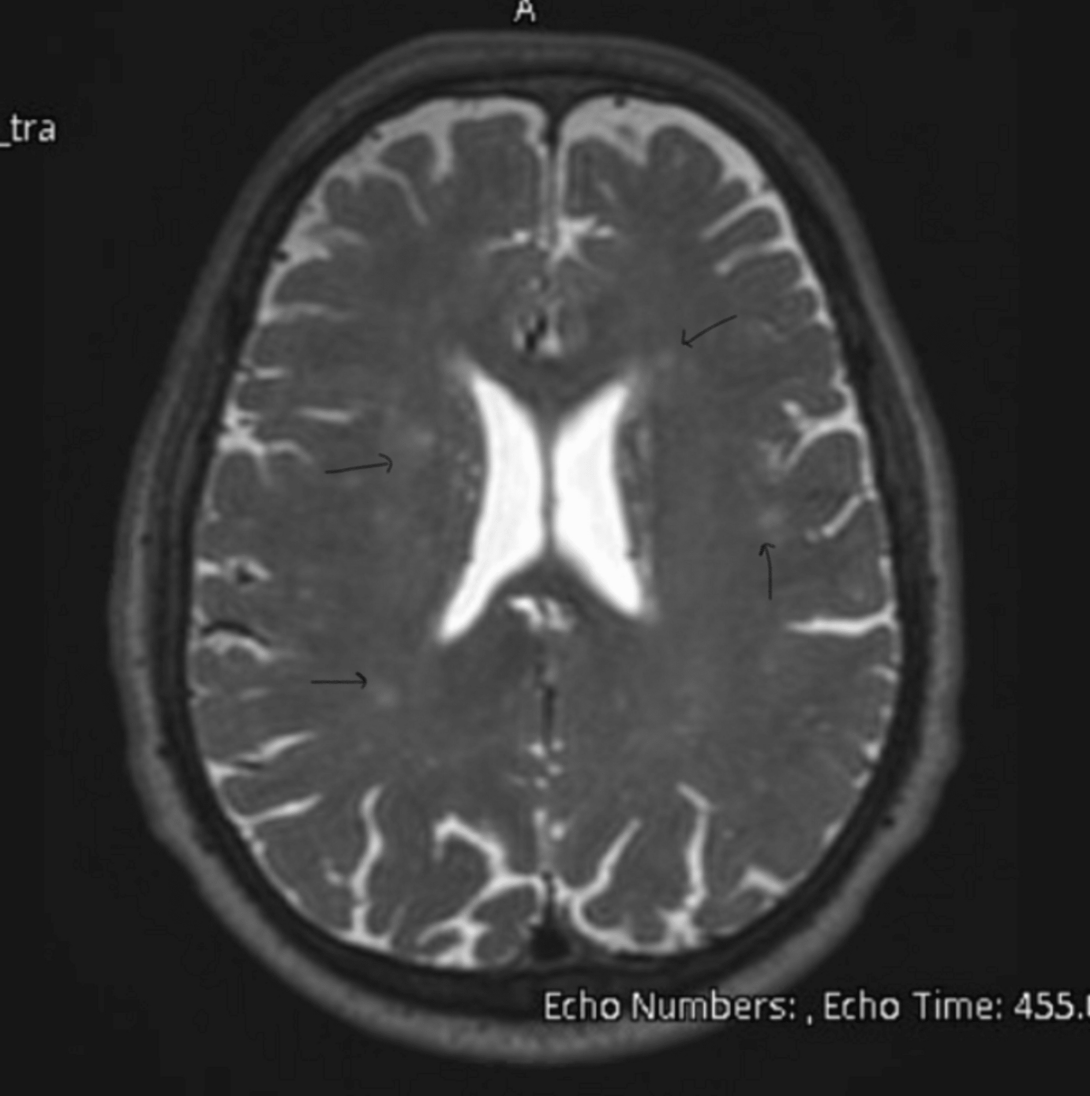
MRI head section 2 showing chronic microangiopathic changes of white matter (black arrows).

An urgent CT head scan was performed, which showed no intracranial abnormalities (Figures 1-2). The neurology team reviewed the case and advised an MRI of the head, which later reported small white matter T2 hyperintensities bilaterally, most likely suggestive of chronic small vessel ischemic changes (Figures 3-4). A routine 24-hour electroencephalogram (EEG) was performed the next day, which showed no epileptiform discharges or interictal epileptiform discharges (IED).

Over the course of two days in the hospital, the patient was seizure-free with no further episodes. He was labeled as having a drug-induced seizure and was discharged home with outpatient follow-up, without antiepileptic medications.

## Discussion

Pseudoephedrine, an over-the-counter sympathomimetic agent, is widely employed for its decongestant properties in the treatment of conditions such as the common cold and allergic rhinitis [1]. The primary mechanism of action of pseudoephedrine involves the stimulation of alpha-adrenergic receptors, leading to vasoconstriction and a reduction in inflammation of the nasal mucosa, thereby relieving nasal congestion [2,4]. Despite its widespread use and effectiveness in the symptomatic treatment of rhinitis and sinusitis, pseudoephedrine has several contraindications. These include hypersensitivity to the drug, cardiovascular diseases (e.g., HTN or coronary artery disease), impaired organ function responsible for drug elimination (severe hepatic dysfunction, moderate to severe renal dysfunction), narrow-angle glaucoma, and benign prostatic hyperplasia [5]. Furthermore, pseudoephedrine can cause a range of adverse effects affecting multiple body systems. Adverse reactions can occur with both oral and intranasal administration, after a single dose or prolonged use (up to five days), regardless of dosage, vascular condition, or age [1].

Central nervous system (CNS) stimulation is among the most commonly encountered adverse effects, manifesting as sleep disturbances, anxiety or restlessness, headache, and confusion [1]. In more severe cases, pseudoephedrine may induce non-convulsive epileptic states in individuals with pre-existing neurological disorders [6]. The risk of complications is exacerbated in patients with impaired renal and hepatic function. Literature review reveals an unusual behavior and myoclonic convulsions were observed in a 64-year-old man with renal failure who ingested 240 mg of pseudoephedrine daily for rhinitis treatment [1].

A 2003 French study documented adverse events associated with intranasal decongestants, reporting 22 episodes of arterial HTN, 15 cases of convulsions, and four cases of stroke following the oral administration of drugs containing pseudoephedrine [3].

The precise mechanism by which pseudoephedrine induces seizures is not fully understood. However, it is hypothesized that pseudoephedrine increases the release of norepinephrine and dopamine in the brain, which may alter neuronal excitability and elevate the risk of seizures [1]. This risk is particularly pronounced in vulnerable individuals, such as the elderly, who may have compromised neurological function or preexisting conditions predisposing them to seizures [5]. Additionally, pseudoephedrine can cause vasoconstriction and increased blood pressure, potentially reducing cerebral blood flow, leading to ischemia, and increasing the risk of seizures [3]. Furthermore, pseudoephedrine and other sympathomimetics may provoke seizures through interactions with medications that lower the seizure threshold. These include certain antidepressants (e.g., monoamine oxidase inhibitors (MAO inhibitors), tricyclic antidepressants), some antihypertensives, proton pump inhibitors, digitalis glycosides, and other sympathomimetics [1].

Other common adverse effects include digestive tract dysfunctions such as nausea, vomiting, and decreased appetite [7,8]. Pseudoephedrine also has stimulant effects on the cardiovascular system, increasing heart rate and elevating blood pressure. Serious cardiovascular side effects, including acute coronary syndrome and hypertensive crisis, have been reported, as in the case of an 87-year-old male heavy smoker who performed strenuous physical work prior to the incident [9].

We report the case of an elderly male with a history of diabetes managed with oral hypoglycemic agents and no prior neurological disorders. The patient presented to the ED with symptoms indicative of a seizure. Prior to the seizure, the patient had self-administered a combination of cetirizine and pseudoephedrine to manage common cold symptoms. The differential diagnosis for a first-onset seizure in an adult is extensive, encompassing metabolic disturbances, structural brain abnormalities, and infectious etiologies. However, in this case, a normal metabolic panel, benign brain imaging, and the absence of fever or focal neurological deficits effectively ruled out these major causes. The temporal association between the onset of the seizure and the use of pseudoephedrine strongly suggests the medication is the likely precipitant.

The patient's outpatient follow-up and the cessation of further seizure activity three months after discontinuing pseudoephedrine further implicate this oral decongestant as the causative agent.

## Conclusions

This case emphasizes the need for vigilant prescribing practices in the elderly population, particularly regarding medications with a potential risk of ADRs, such as pseudoephedrine. Recognizing seizure provoked by pseudoephedrine as a possible side effect is crucial given its widespread use as an over-the-counter decongestant. This case underscores the importance of considering pseudoephedrine in the differential diagnosis of seizures, especially in patients with recent exposure to the medication.

Further research is needed to better understand the potential risks and benefits of pseudoephedrine use in the elderly. Additionally, investigations could explore alternative treatments for nasal congestion in elderly patients, such as intranasal corticosteroids or saline irrigation, which may carry a lower risk of adverse effects.
